# Increase in number of circulating disseminated epithelial cells after surgery for non-small cell lung cancer monitored by MAINTRAC^® ^is a predictor for relapse: A preliminary report

**DOI:** 10.1186/1477-7819-3-18

**Published:** 2005-03-31

**Authors:** Axel Rolle, Rainer Günzel, Ulrich Pachmann, Babette Willen, Klaus Höffken, Katharina Pachmann

**Affiliations:** 1 Fachkrankenhaus Coswig/Dresden, der Friedrich-Schiller-Universität Jena, Germany; 2 TZB Transfusionsmedizinisches Zentrum Bayreuth, der Friedrich-Schiller-Universität Jena, Germany; 3 Klinik für Innere Medizin II der Friedrich-Schiller-Universität Jena, Germany

## Abstract

**Background:**

Lung cancer still remains one of the most commonly occurring solid tumors and even in stage Ia, surgery fails in 30% of patients who develop distant metastases. It is hypothesized that these must have developed from occult circulating tumor cells present at the time of surgery, or before. The aim of the present study was to detect such cells in the peripheral blood and to monitor these cells following surgery.

**Methods:**

30 patients treated for lung cancer with surgery were monitored for circulating epithelial cells (CEC) by taking peripheral blood samples before, 2 weeks and 5 months after surgery and/or radiotherapy (RT) chemotherapy (CT) or combined RT/CT using magnetic bead enrichment and laser scanning cytometry (MAINTRAC^®^) for quantification of these cells.

**Results:**

In 86% of the patients CEC were detected before surgery and in 100% at 2 weeks and 5 months after surgery. In the control group, which consisted of 100 normal donors without cancer, 97 % were negative for CEC. A significantly higher number of CEC was found preoperatively in patients with squamous cell carcinoma than in those with adenocarcinoma. In correlation to the extent of parenchymal manipulation 2 weeks after surgery, an increase in numbers of CEC was observed with limited resections (18/21) whereas pneumonectomy led to a decrease (5/8) of CEC, 2 weeks after surgery. The third analysis done 5 months after surgery identified 3 groups of patients. In the group of 5 patients who received neo- or adjuvant chemo/radiotherapy there was evidence that monitoring of CEC can evaluate the effects of therapy. Another group of 7 patients who underwent surgery only showed a decrease of CEC and no signs of relapse. A third group of 11 patients who had surgery only, showed an increase of CEC (4 with an initial decrease after surgery and 7 with continuous increase). In the group with a continuous increase during the following 24 months, 2 early relapses in patients with stage Ia adenocarcinoma were observed. The increase of CEC preceded clinical detection by six months.

**Conclusion:**

We consider, therefore, that patients with adenocarcinoma and a continuous increase of CEC after complete resection for lung cancer are at an increased risk of early relapse.

## Background

Lung cancer still remains the most frequent solid tumour in men and represents the leading cause of death in women since the 1980s in the USA [1]. Due to a steady increase in cigarette smoking, even in Europe and Germany, lung cancer has become three times more common in women during the last 20 years [2].

Despite good progress in diagnosis, operative and multimodality treatment of patients with lung cancer the prognosis has remained persistently bad during the last 50 years, with 5 years survival rates of 9% for all patients [3]. The therapeutic dilemma becomes evident if results from stage Ia lung cancers are examined. If patients undergo a complete resection, the therapy of choice, then the 5 year survival is 70%. But treatment has failed for the 30% of patients who develop distant metastases in the two years following surgery [4]. The metastases in these patients must have developed from occult circulating tumor cells present at the time of their original surgery, or before, leading to an underestimation of the true tumor stage. Pantel et al [5] detected epithelial cells in 20–25 % of biopsies taken intra-operatively from bone marrow after resection of lung cancers. Nevertheless, even today the role of cytokeratin positive cells in bone marrow remains unclear. They may have come from the tumor at an early stage, or seeded into the circulation during surgery. lt is also not clear whether these cells have proliferative activity or are so-called dormant cells [6].

Using a method for rapid determination of circulating epithelial cells (CEC) in peripheral blood [7], which allows monitoring of these cells under therapy, we demonstrate that a single examination is not sufficient to determine outcome. However, it is necessary to monitor the behavior of these cells over time to be able to predict tumor recurrence.

## Methods and methods

Anti-coagulated peripheral blood samples were drawn after informed consent from a control group of 100 normal subjects aged between 17 and 73 years and from 30 successive patients before, and two weeks after, surgery for lung cancer. A third sample was obtained 3 to 5 months postoperatively when the patients were seen for follow-up. In all patients routine controls were continued up to 27 months or death with a median follow-up of 22 months.

Seven patients were excluded. In one patient lung cancer was not confirmed after histological examination and in another patient lung cancer was combined with advanced sarcoidosis. In 5 patients one or two samples were missing therefore these cases were omitted as well, despite there being no further discrepancy. Of the remaining 23 patients, for which all data and follow-up were complete, 20 were male (mean age 64 years, range 55 – 74 years) and 3 were female (mean age 53 years, range 40 – 74 years) with a balanced proportion of early to advanced stages of lung cancer (12:11). Staging was performed by chest x-ray, endobrochial bronchoscopy, computerized tomography (CT), bone scan, operative lymph node dissection and size of tumor-free margin. Further information about surgery, histological type of tumor stage and the number of CEC is depicted in table 1.

**Table 1:** Data from 23 patients operated for lung cancer

						**CEC Levels**					
**No**	**age**	**sex**	**tumor histology**	**operation**	**Pathologic stage**	**1^st ^analysis pre op**	**2^nd ^analysis 2 weeks pop**	**3^nd ^analysis 5 month pop**	**Change**	**therapy**	**follow up**

1	55	M	squamous	pneumonectomy	III a	769.091	664.210	853.333	⇒	neoadj. CT	Ø R 24 months
2	62	M	squamous	pneumonectomy	II b	0	474.375	5.722.667			Ø R 27 months
3	68	M	squamous	bilobectomy	I b	5.396.667	2.222	65.000			Ø R 25 months
4	68	M	squamous	pneumonectomy	III a	1.514.583	80.000	35.185			Ø R 25 months
5	67	M	squamous	pneumonectomy	I b	292.592	292.592	1.312.000			Ø R 26 months
6	57	M	squamous	lobectomy	I b	64.942	476.667	610.000			Ø R 26 months
7	68	M	adeno	pneumonectomy	III a	1.000.000	110.000	96.429	⇒	adj. RT	T † 26 months
8	66	M	squamous	pneumonectomy	I b	0	5.000	203.333			Ø R 25 months
9	56	M	adeno	lobectomy	I a	224.118	615.556	1.656.000			R † 24 months
10	74	M	squamous	explorative	III b	127.059	147.000	70.000		adj.CT	T alive 25 months
11	60	M	adeno	lobectomy	I b	3.158	203.333	13.889			Ø R 22 months
12	61	M	adeno	lobectomy	I b	28.500	6.667	46.667			Ø R 25 months
13	41	F	adeno	lobectomy	I a	20.625	51.667	98.333			R † 11 months
14	67	M	squamous	pneumonectomy	II b	16.667	9.259	93.333			Ø R 14 months
15	69	M	adeno	segmentectomy	I b	6.667	25.000	100.000			Ø R 24 months
16	68	M	adeno	explorativ	III b	71.667	140.000	263.571		adj. CT/RT	T † 9 months
17	65	M	adeno	lobectomy	I b	0	335.000	182.857			Ø R 13 months
18	71	M	adeno	lobectomy	I b	22.941	17.778	1.605.185			Ø R 23 months
19	68	M	adeno	lobectomy	III a	6.667	57.647	11.111		adj. RT	Ø R 23 months
20	66	M	adeno	lobectomy	I a	126.667	154.286	38.571			Ø R 22 months
21	57	M	squamous	pneumonectomy	I b	473.333	1.031.250	503.333			Ø R 23 months
22	74	F	squamous	lobectomy	II b	11.053	160.000	150.000	⇒		Ø R 19 months
23	56	F	adeno	segmentectomy	I b	5.000	2.670.370	576.190			Ø R 23 months

CT = Chemotherapy, RT = Radiotherapy, R = Recurrence, ∅ R = no Recurrence, T = Tumor, T † = died with tumor, adj. = adjuvant, neoadj. = neoadjuvant, Pop = Postoperatively, Adeno = Adenocarcinoma, Squamous = Squamous carcinoma

### Cell preparation

Red blood cells from 10 ml samples of blood were lysed, the white blood cells spun down and epithelial cells were enriched using micro-beads from Miltenyi (Bergisch Gladbach, Germany) [7] or the method by Labsoft Diagnostics (Halle, Germany) [8]. Both methods yielded comparable results. The same method was always used in follow-up samples from the same patient. Cells were stained with FITC conjugated mouse anti-human epithelial antibody (HEA, Milteny, Bergisch Gladbach, Germany), reacted either with HEA-micro-beads Miltenyi (Bergisch Gladbach, Germany) or Cell Select-micro-beads (Labsoft Diagnostics Halle, Germany) and then either separated over the columns provided by the Miltenyi company according to their description or the vials containing the cells were applied to magnets provided by the Labsoft company. Negative cells not bound in the columns were washed out, the columns removed from the magnet and the retained cells eluted. In the Labsoft method cells not attached to the vial wall were sucked off, the vials removed from the magnet and the cells retained in the vials were then flushed off the wall and studied.

### Analysis

For measurements the cells were applied to adhesion slides (Menzel, Gläser, Germany) and measurements started when the cells had settled using a Laser Scanning Cytometer (LSC^® ^Compucyte Corporation, Cambridge, MA, USA). The cells could easily and unequivocally be contoured using forward scatter as a threshold parameter. Background fluorescence was determined dynamically to calculate both peak and integral fluorescence on a per-cell basis making the fluorescent calculation equivalent for all cells. The green fluorescence was displayed as scattergrams and histograms.

**Figure 1:**
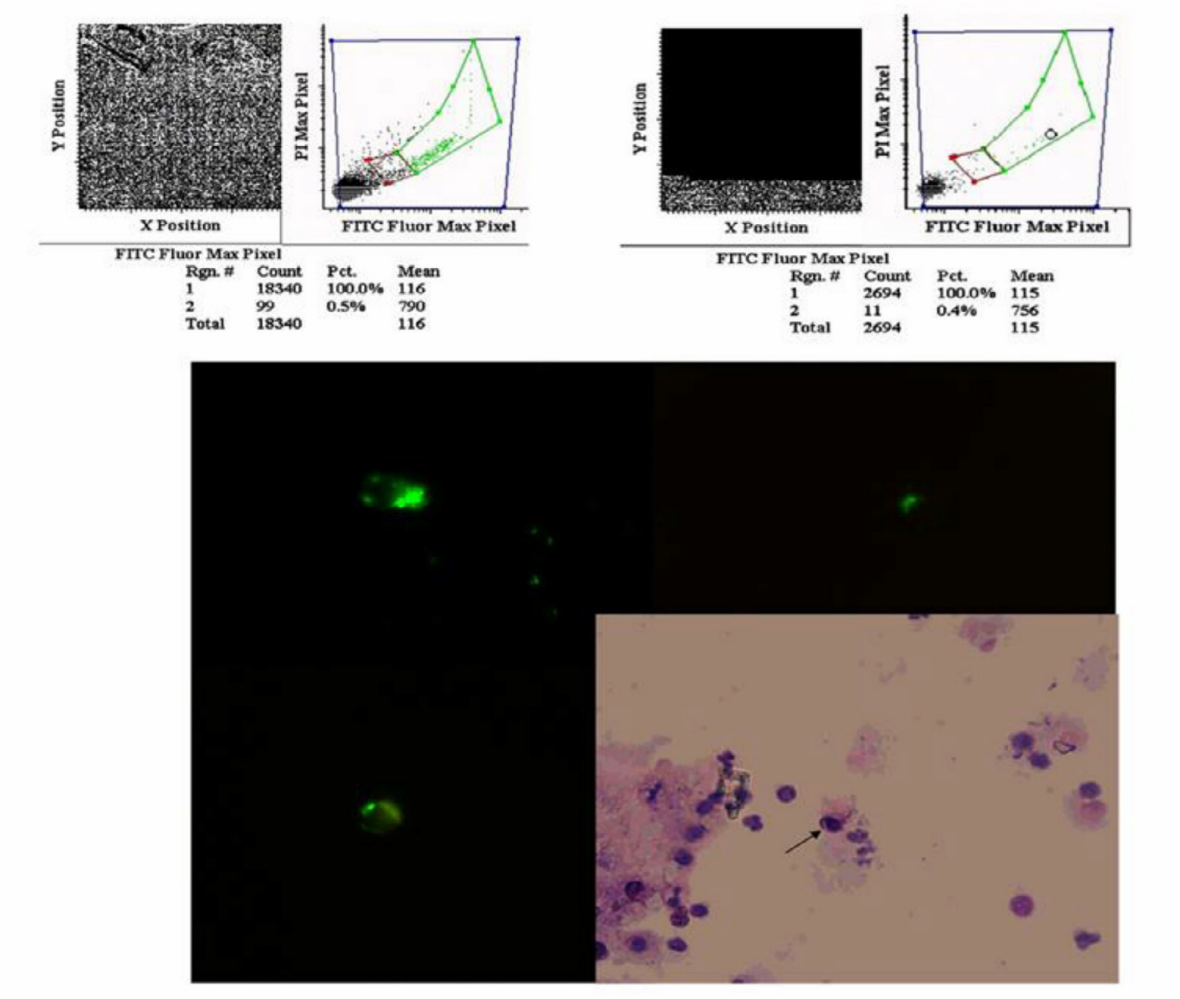
Example of an analysis of a slide with epithelial antigen positive and negative cells: a) fluorescence intensity displayed as a dot plot with all cells in region 1 and the positive cells gated in region 2 (green region). b) The scanning stage proceeds from one positive event to the next stopping at each position of a positive event and allowing the observer to control whether it represents a specific membrane stained cell as shown in figure 1c. c) Three typical cells shown with green fluorescence. In the lower panel the identical cell is shown in panoptic staining indicated by the arrow.

The LSC^® ^enables the user to relocate cells contained within the positive population for visual examination through the microscope (Figure 1). In addition a CCD camera attached to the microscope allowed taking photo- and fluoromicrographs at the same time. More detailed morphology could be determined in conventional panoptic staining (Figure 1).

## Results

**Figure 2:**
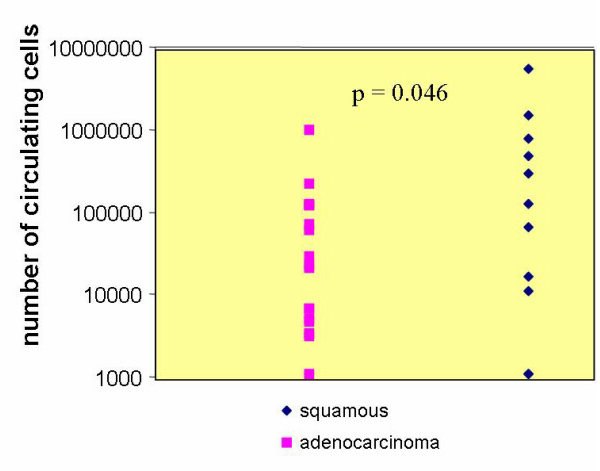
Number of preoperative circulating tumor cells in comparison to histological classification.

**Figure 3:**
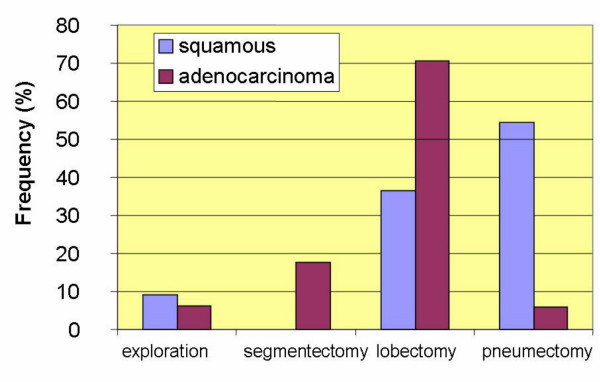
Frequency of surgical interventions in comparison to histological classification.

Circulating epithelial cells (CEC) were detectable in blood samples from 25/29 patients (86%) before surgery, whereas 97% of normal controls were negative for these cells. The number of CEC was calculated in relation to the analyzed blood volume and not to white blood cells, which vary considerably during the course of disease. For 26 patients histological evaluation was available and revealed 15 cases of adenocarcinoma and 11 cases of squamous cell carcinoma. The comparison of preoperative numbers of CEC revealed higher values in patients with squamous cell than in those with adenocarcinoma and these values differed significantly (p = 0.046) (Figure 2). Neither tumor size nor positivity of lymph nodes correlated with the number of circulating tumor cells before surgery. With regard to the extent of resection 1 pleurectomy, 8 pneumonectomies, 1 bilobectomy, 12 lobectomies, 4 segmental resections and 3 explorative thoracotomies were performed. Lobectomy was more frequent than pneumonectomy in patients with adenocarcinoma (69% vs. 36%) and pneumonectomy more frequent than lobectomy in those with squamous cell carcinoma (54% vs. 6%) (Figure 3).

**Figure 4:**
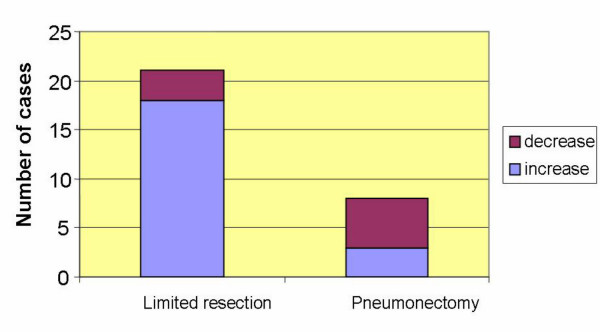
Changes in cell numbers (increase or decrease) following surgical intervention.

**Figure 5:**
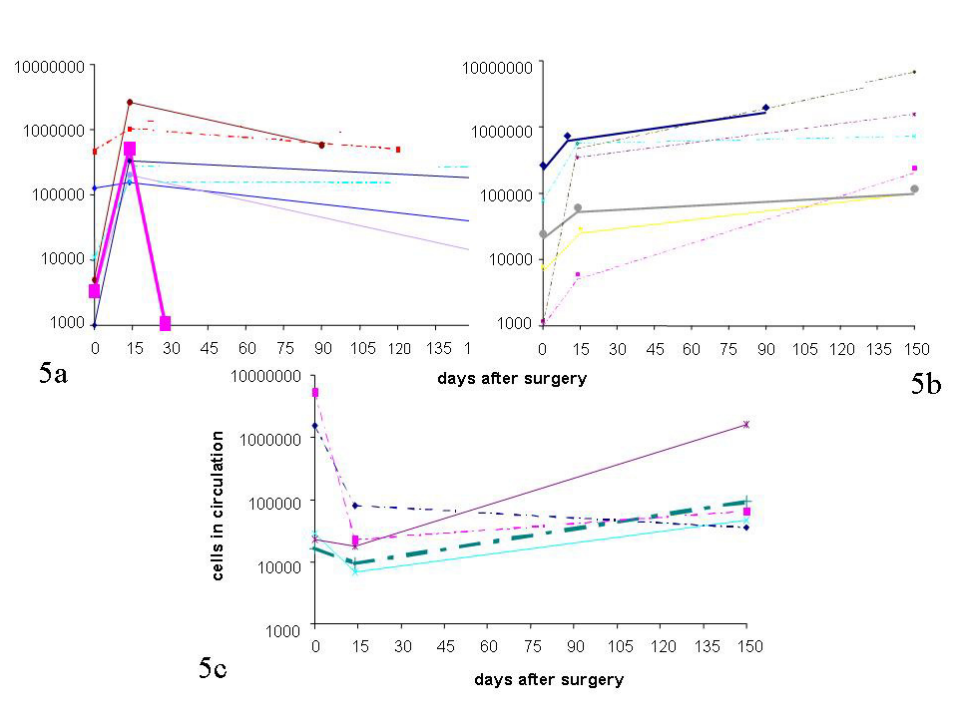
Trends of CEC numbers in individual patients after complete resection without further treatment: a) patients with an increase in CEC 14 days after surgery and a subsequent decrease during the next five months (dotted lines: squamous cell cancer; solid lines: adenocarcinoma; fat line and squares: patient with benign lung disease pneumothorax) b) patients with an increase in CEC 14 days after surgery and a subsequent further increase during the next five months (dotted lines: squamous cell cancer; solid lines: adenocarcinoma; fat lines: relapsed patients) c) patients with a decrease in CEC 14 days after surgery (dotted lines: squamous cell cancer; solid lines: adenocarcinoma)

Fourteen days postoperatively, CEC were detected in all patients including the 3 where no cells had been detectable before surgery. In cases with limited resection (3 exploratory thoracotomies, 4 segmental resections, 12 lobectomies, 1 bilobectomy) parenchymal manipulation was more extensive. This resulted more frequently in an increase (85.7%) than in a decrease (14.3%) in CEC, whereas in cases of complete resection of the lung (pneumonectomy) numbers of CEC decreased (62.5%) more frequently than increased (37.5%) (Figure 4). We, also, investigated CEC in one patient who had surgery for pneumothorax and observed a comparable increase in CEC as in the tumor patients but with a rapid re-decrease already after another two weeks (Figure 5a).

A third analysis of CEC was performed between 3 and 5 months after surgery. In 23 (76%) of 30 consecutive patients considered to have a resectable non small cell lung cancer (NSCLC) complete data were available with a median follow-up of 22 months (range 9 – 27 months) (Table 1).

Five out of 23 patients underwent chemotherapy (CT) or chemo/radiotherapy (CT/RT). These patients were considered separately from the 18 patients who had complete curative resection of their tumors without adjuvant treatment. Two patients underwent exploratory thoracotomy: one with squamous cell carcinoma (patient 10) was treated with adjuvant CT with a subsequent decrease in CEC and is still alive 25 months after surgery. The other patient with adenocarcinoma (patient 16) showed an increase in CEC in spite of combined RT/CT and died 9 months later with tumor progression.

Three of the 21 patients with tumor resection received additional treatment: 1 lobectomy patient with adenocarcinoma had N2 lymph node status (patient 19). This patient with one level N2 status received adjuvant RT. He subsequently showed a decrease in CEC and is alive, 23 months after surgery without signs of relapse. One patient with squamous cell carcinoma (patient 1) received neoadjuvant therapy and subsequently was treated with pneumonectomy. He showed a slight increase in CEC but is still alive without signs of relapse. Another patient with adenocarcinoma (patient 7) underwent RT but with no significant decrease in CEC and died 26 months later with tumor progression. Among these patients receiving additional radio- and/or chemotherapy relapses occurred only in those with adenocarcinoma and increasing CEC numbers in spite of treatment.

Of the remaining 18 patients who had complete curative resection of their tumors, 7 showed a decrease, and 11 showed an increase in CEC during the 3–5 months follow-up after surgery.

Of the former 7 patients 6 had had cell numbers increasing after surgery and subsequently decreasing during the following months (patients 11, 17, 20, 21, 22, 23), 4 with adeno- and 2 with squamous cell carcinoma and all but one (patient 21) had lobectomy (Figure 5a). All are in complete continuous remission. 1 patient (patient 4) with squamous cell carcinoma had pneumectomy and continuously decreasing cell numbers (Figure 5c) and he, too, is in complete continuous remission.

Among the 11 patients with increasing CEC there were 4 with an initial decrease after surgery followed by a subsequent increase (Figure 5c). None has shown early relapse so far. Seven had a continuous increase from pre-surgery to 5 months after treatment (Figure 5b), 4 with squamous cell carcinoma and 3 with adenocarcinoma. Two relapses were observed amongst these patients during the subsequent 2 years of follow-up. Both, a male (56 years of age) and a female (41 years of age), had adenocarcinoma stage Ia. They had continuously increasing CEC and the increase occurred six months before repeated clinical examinations could verify disease relapse. Both received chemotherapy after diagnosis of relapse but died despite 9 and 24 months after surgery with systemic spread of their tumor.

In summary, amongst the patients with adjuvant treatment after resection, as well as in curatively resected patients, patients with adenocarcinoma and a continuous increase in CEC during the course of their disease have a significantly higher risk of early relapse than those with decreasing cell numbers and than those with squamous cell carcinoma, even if the latter show increasing numbers of CEC.

In the relapsed patients not single values of circulating tumor cells at different time points but only the increase in cell numbers was a predictor of relapse.

## Discussion

Analyzing circulating epithelial cells in peripheral blood from patients with non small cell lung cancer, we were able to detect live CEC in 86% of patients before surgery. This represents a significantly higher number and frequency than reported from other groups analyzing micrometastases taken from bone marrow [9,10] but cell numbers are in the same range as those reported by Cristofanilli and coworkers [11]. This discrepancy may be explained by several modifications of pre-analytical handling: 1) In contrast to the former authors [9,10] we used red blood cell lysis [7] for white blood cell preparation. During ficoll-separation used for preparation of cells from bone marrow aspirates we observed a loss of up to 90% epithelial antigen positive cells and there is no reason why circulating epithelial cells should have the same density properties as mononuclear cells (Pachmann submitted for publication]. 2) Micrometastatic cells can be hidden in the fat lumps of the bone marrow aspirates and thus be lost for analysis when preparing mononuclear cell suspensions. 3) The portion of blood admixed to marrow aspirates can vary between 10 and 90% resulting in a high variability of the material used for analysis. Although the method applied by us is also suitable for bone marrow samples, we preferred analysis of peripheral blood samples which are easy to obtain and can be drawn repeatedly.

The number of CEC detected by us in lung cancer patients is somewhat lower than the number reported recently by Cristofanilli *et al *[11] in metastatic breast cancer patients. In positive patients we found between 2000 and 6 × 10^6 ^epithelial cells calculating the number of cells in the circulation assuming that a patient has about 5 liters of blood. The values of 5 to 22000 cells per 7.5 ml blood reported by Cristofanilli *et al *[11] would translate into 3000 to 13 × 10^6 ^cells in the circulation. The frequency of preoperatively CEC positive patients was comparable to that reported by Racila *et al *[12] and Krag *et al *[13] who also used a similar method for analyzing blood from breast cancer patients.

We preferred detection of fluorescent antibody bound to the surface of live epithelial antigen positive cells performed by an automated Laser Scanning Cytometer LSC^® ^to PCR based methods. PCR methods are currently regarded as the most sensitive procedures, but there is a reported high frequency of false positives [14-16]. In addition, our procedure allowed subsequent separation and even culturing of the isolated cells. Culturing will definitely allow determination whether the cells are dormant or have the potency of differentiating into tumor cells.

It should be mentioned that we observed significantly higher numbers of CEC preoperatively in patients with squamous cell carcinoma than in those with adenocarcinoma. This could be due to the former being more centrally located (next to the great vessels) which might lead to a higher dissemination than occurs in more peripherally located tumors.

Two weeks after surgery all patients were positive for CEC, but there was no correlation to stage, tumor size or lymph node involvement. Unexpectedly we found a strong correlation between postoperative increase and limited resections (segmental resections and lobectomies) and a decrease of CEC after pneumonectomies. We can only explain this observation by the extended parenchymal manipulation involving the separation of parenchymal bridges between lobes and late ligation of pulmonary vessels during lobectomies or segmentectomies. In contrast, pneumonectomies normally start with ligation of the vessels and subsequent resection of the lung. Perhaps our data confirm once more the importance of careful "No touch" preparation during oncological resections [17].

However, during parenchymal resections also, normal epithelial cells from the lung can be mobilized and washed out entering the vessels. This may be true in the cases where we observed a reduction of CEC during the following six months of observation. Our attempts to differentiate benign isolated epithelial cells from malignant cells by their morphology failed, and molecular biological techniques will need to be applied [18,19] to solve this problem. But generally the premise is accepted, that disseminated normal cells will die in a short time and only the portion of cells that are malignant will have a chance of survival for unknown periods of time. [18]. Monitoring the CEC after five months would, therefore, be expected to detect only surviving malignant epithelial cells. Once again we found CEC in all patients.

Analysis of the increase or reduction of CEC in the 5 patients receiving neoadjuvant and/or adjuvant CT and RT due to their nodal status indicated that this parameter can possibly become a tool for monitoring the effectiveness of therapeutic interventions, an effect that we are actually investigating now in patients receiving CT for lung cancer.

Of the 18 patients treated with complete curative resection all 7 patients of the group with decreasing numbers of CEC were without signs of relapse up to 27 months after surgery. In the group of 11 patients with increasing numbers of CEC, 6 had squamous cell carcinomas and none has relapsed during the observation time of 2 years in accordance with published data [20]. In contrast, in the group of adenocarcinomas 2/5 early relapses were observed, both in patients with stage Ia with the increase in CEC preceded overt clinical relapse by 6 months.

## Conclusion

Thus, postoperative monitoring of CEC may become a reliable prognostic predictor of relapse. The observation that only 20% of the patients in the group at risk develop early relapse confirmed by other studies [21] and that patients with squamous cell carcinomas can have high and over time even increasing numbers of CEC necessitate additional analyses on the CEC to obtain more information about their metastatic potential [22]. Such analyses on enriched and cultured isolated CEC are now under way in our laboratory.

## Competing interests

The author(s) declare that they have no competing interests.

## Authors' contributions

**AR: **carried out the studies, and drafted the manuscript

**RG: **conceived of the study, and participated in its design and coordination

**UP: **participated in the design of the study and performed the statistical analysis

**BW: **carried out the immunoassays

**KH: **preparation of the manuscript

**KP: **participated in the design of the study and performed the statistical analysis

All authors read and approved the manuscript

## Research grant

Not declared
